# Supplementary material for: Surgical decision making in the setting of severe traumatic brain injury: A survey of neurosurgeons
Source: PLoS One. 2020 Mar 2;15(3):e0228947. doi: 10.1371/journal.pone.0228947 (PMC7051065; doi:10.1371/journal.pone.0228947)
Supplement: S1 Survey — (DOCX) [file pone.0228947.s001.docx]

**Critical Neurotrauma Decision Making Survey**

As a junior resident on call, I became familiar with the scenario of calling attending neurosurgeons at all hours to discuss cases of brain trauma, seeking advice on prognosis and thoughts about whether or not to intervene surgically. I used their input to guide difficult conversations with patient families. Being struck by the complexity and difficulty of such decisions, I decided to conduct a study to better understand how experts address incredibly challenging discussions with families based on limited clinical and prognostic information.

The following scenarios represent calls to attendings from the emergency room trauma bay about patients just arriving. ***Please read the cases as though they are phone calls from a junior resident*** like myself and you are the attending neurosurgeon on call and use the available, albeit limited, information to answer the questions below.

All answers are confidential. You may stop the survey or ask questions about it at any time. Thanks for your help!

*____________________________________________________________________________*

*First, please answer a few questions about you:*

1. Medical Specialty______________
2. Gender ______________
3. Race/Ethnicity ______________
4. Year of Birth ______________
5. What is your religion, if any? ______________
6. Year of medical school graduation ______________
7. In which country is your primary practice? ______________
8. In which state do you practice? ______________

Please check one for each question below:

1. Level of training  intern  resident  fellow attending
2. Do you supervise residents/fellows?  Yes  No
3. How important is religious faith in your life?

Very important Important Neutral Unimportant Very unimportant

1. Which best describes your current employer (Check one)?

Independent, physician-owned practice

employed by a university hospital/health system

employed by a non-university hospital/health system

1. Is your principal practice site a level 1 trauma center?  Yes  No

**🕿 You receive a phone call about…**

A previously independent 77-year-old male who presents following a mechanical fall down stairs with isolated head trauma, found down with unknown duration by his family.

**PMH:** Unknown, no known medications.

**Exam:** he is not opening eyes or talking but flexion posturing, and both pupils are reactive. Blood pressure and oxygen saturations are within normal limits. No obvious extra-cranial injuries.

**CT brain:** demonstrates a 1 cm acute subdural hematoma with 1 cm of midline shift. There is also scattered traumatic subarachnoid hemorrhage.

**Labs:** glucose is 90mg/dL, coagulation studies within normal limits, and hemoglobin is 9g/dL.

After describing the patient to you, the resident tells you about a prognostic estimate from a tool called “the CRASH calculator.” The calculator uses data from prior studies in severe traumatic brain injury to create a positive predictive value of a factor’s effect on mortality. It is not meant to substitute clinical judgment however is intended to support it. According to the CRASH calculator, **with the hematoma evacuated**, this patient faces a:

- **14-day mortality risk** 65.6%
- **6-month unfavorable outcome risk** 93%

(death, vegetative state, or severe disability)

The junior resident on call asks your guidance on the following questions:

1. What is the best next step (Check 1 box)?

Take to the OR

Admit to the ICU for aggressive medical management

Initiate comfort care measures

2. Suppose the family insists upon surgical intervention. Because the CRASH estimates do not substitute clinical judgement, you’re asked for your best prognostic guesses. If you proceed with surgery, what is your best guess, number of patients out of 100 that will…

Best Guess (0-100%)

a. _____% survive the hospitalization?

b. _____% survive to 6 months?

c. _____% survive to 30 days?

d. _____% Be able to communicate and perform activities of daily living

(ADLs) within the next 6 months?

e. _____% live permanently in a nursing home?

f. _____% rely permanently on a ventilator?

Comments: _______________________________________________________

**🕿 You receive a second call about…**

A 60-year-old man presents following a fall with obvious head trauma, found down with unknown duration by his family.

**PMH:** Unknown, no known medications.

**Exam:** he is unable to speak or open his eyes and is symmetrically extending all extremities. Both pupils are non-reactive. Blood pressure and oxygen saturations are within normal limits. No obvious extra-cranial injuries.

**CT scan** demonstrates a 1 cm subdural hematoma with 1 cm of midline shift with traumatic subarachnoid hemorrhage.

**Labs:** His glucose is 90mg/dL and hemoglobin is 9g/dL. Coagulation studies are within normal limits.

According to the CRASH calculator, **with the hematoma evacuated**, this patient faces a:

- **14-day mortality risk** 73.1%
- **6-month unfavorable outcome risk** 93%

(death, vegetative state, or severe disability)

The junior resident on call asks your guidance on the following questions:

1. What is the best next step (Check 1 box)?

Take to the OR

Admit to the ICU for aggressive medical management

Initiate comfort care measures

2. Suppose the family insists upon surgical intervention. Because the CRASH estimates do not substitute clinical judgement, you’re asked for your best prognostic guesses. If you proceed with surgery, what is your best guess, number of patients out of 100 that will…

Best Guess (0-100%)

a. _____% survive the hospitalization?

b. _____% survive to 6 months?

c. _____% survive to 30 days?

d. _____% Be able to communicate and perform activities of daily living

(ADLs) within the next 6 months?

e. _____% live permanently in a nursing home?

f. _____% rely permanently on a ventilator?

Comments: _______________________________________________________

**(turn page)**

| **How much do you agree/disagree with the following statements (specific to severe TBI)?** | Strongly Agree | Agree | Neutral | Disagree | Strongly Disagree |
| --- | --- | --- | --- | --- | --- |
| Looking back on patients with severe TBI, I have **recommended** surgery in patients that I should not have. |  |  |  |  |  |
| Looking back on patients with severe TBI, I have **not recommended** surgery in patients that I should have. |  |  |  |  |  |
| I am responsible if a patient dies within 30 days of any surgery I recommend. |  |  |  |  |  |
| I am responsible if a patient survives in a state they would not find acceptable after a surgery I recommended |  |  |  |  |  |
| As a physician, I am willing to **not offer** surgery even if it means a patient will die. |  |  |  |  |  |
| Regardless of prognosis, I am obligated to offer surgery if there is a chance the patient will survive. |  |  |  |  |  |
| I worry about incorrectly estimating prognosis. |  |  |  |  |  |
| I use available literature and data to make a prognosis. |  |  |  |  |  |
| I use prior experience with patients to make a prognosis. |  |  |  |  |  |
| I feel that there is adequate quality data to base prognostication. |  |  |  |  |  |

| **How would you rate the following health states?** | Much better than death | Somewhat better than death | Little bit better than death | Neither better nor worse than death | Worse than death |
| --- | --- | --- | --- | --- | --- |
| Bowel and bladder incontinence |  |  |  |  |  |
| Rely on breathing machine to live |  |  |  |  |  |
| Cannot get out of bed |  |  |  |  |  |
| Confused all the time |  |  |  |  |  |
| Rely on feeding tube |  |  |  |  |  |
| Need 24-hour care |  |  |  |  |  |
| Living in nursing home |  |  |  |  |  |

Comments: _______________________________________________________
