# Supplementary material for: Surgical decision making in the setting of severe traumatic brain injury: A survey of neurosurgeons
Source: PLoS One. 2020 Mar 2;15(3):e0228947. doi: 10.1371/journal.pone.0228947 (PMC7051065; doi:10.1371/journal.pone.0228947)
Supplement: Attachment — Submitted filename: Response to Reviewers.docx [file pone.0228947.s002.docx]

Response to Reviewers –  PONE-D-19-24519

We thank the editors and reviewers for their thorough review and for providing constructive critique on the manuscript. Please find below a detailed point-by-point response to the reviewers’ concerns.

**Reviewer #1**

**Overall**

The two scenarios are essentially treated as two separate questions for much of the paper, which brings up the point of multiple comparisons. Either the second scenario is a “control” which the authors did not anticipate would vary with providing prognostic data, or the issue of multiple comparisons needs to be addressed/taken into account appropriately during analyses for significance. Alternatively, the authors could have performed analyses while somehow removing/regressing out the effect of the patient scenario to see the global effect across the entire study. Either way, the possibility of performing multiple comparisons needs to be addressed.

We thank the reviewer for pointing out that we were not sufficiently clear about this. In fact, as stated in the Methods section – “*The experiment was powered on the effect of evidence-based risk estimates on craniotomy recommendation”* – we powered the study on the binary craniotomy recommendation outcome only. Furthermore, since the two scenarios are similar and the response to the second scenario is subject to anchoring effects from the first scenario, we did not find it meaningful to treat them as independent hypotheses. In consequence, we had only powered the study on craniotomy recommendation in the first scenario. We now explicitly state this in the updated methods section:

*“The experiment was powered on the effect of evidence-based risk estimates on craniotomy recommendation in the first scenario.”*

In the revised manuscript, and following the ASA recommendations for the use of p-values, we now

- We provide the p-value only for the craniotomy recommendation in scenario 1 (for which we powered the study)
- We omit “statistically significant” terminology throughout the article
- We use effect sizes and confidence intervals without p-values for other outcomes

Following our initial pilot study, we decided to include a second scenario to test if a different clinical picture would result in a different response from neurosurgeons. This still warrants further study. Our study showed a possible effect of CRASH in 1 specific scenario that we conclude is part of a multifactorial decision process and does not generalize to other studies, particularly the second scenario included in our study.

**Abstract**

Methods – Please more clearly explain the groups in methods, else the results section is less clear to the reader.

We have updated this as follows

*In a survey-based randomized experiment, a total of 138 neurosurgeons were presented with two hypothetical patients with severe TBI and subdural hematoma; the intervention group received additional evidence-based risk estimates for each patient. The main outcome was neurosurgeon treatment recommendation of non-surgical treatment. Secondary outcomes included prediction of functional recovery at six months.*

Results – As written it is not clear that the surgery recommendation changing is only for one of the two groups.

We have addressed by explicitly reporting the results for scenario one, and adding the following comment:

- The results for the second patient scenario were qualitatively similar.

**Paper**

194 – The effect was borderline and not a significant effect, therefore one can claim it at most as a trend only.

As per our response to the first concern above, we now follow ASA recommendations and abstain from such language altogether.

195 – Is the mediation analysis for both scenarios or just the 1st ?

We clarified this as follows at the end of the subsection:

In scenario two, a mediation analysis was not warranted because provision of evidence-based risk estimates had no effect on treatment recommendation.

203 – The results of a multivariable analysis are less convincing when independent variable A (6-month ADL) is relatively correlated with variable D (whether the participant received CRASH data). There seems to be a decent degree of multicollinearity between 6-month ADL and test version, which depending on the strength may be an argument against performing this particular analysis at all. Did the authors validate the use of multiple linear regression by testing Variance Inflation Factor values?

We are not quite sure that we fully understand the reviewer’s concern here. In our opinion, correlation between 6-month ADL and test version is actually needed to justify a mediation analysis in the first place. Furthermore, the comparison of regression of treatment decision on risk estimate provision both with and without adjusting for the potential mediation seems to be critical for supporting evidence of mediation.

Regarding the last point: as stated in the title of this subsection, this is an exploratory analysis only. While we agree with the reviewer that variance inflation analyses would be desirable for a definitive mediation analysis, we believe that they may lead to overconfidence in what is meant to be an exploratory analysis here.

**Figures and Tables**

Table 2 – In the trauma center hospital section the number and percent data don’t make sense. I think the percent data in row “other” for control and intervention columns should be in the “Level 1” row AND the number data for intervention “other” should be in the level 1 row.

We double checked and it should now be correct.

**Reviewer #2**

The authors present a survey analysis from a random convenience sample of 139 neurosurgeons from the Congress of Neurological Surgeons (CNS) meeting 2017. The goal of the study was to evaluate surgical decision making in the setting of severe TBI and to determine the impact of availability of evidence-based risk estimates on prognostication and treatment recommendation. The authors found that prognostic beliefs and surgical recommendation were highly variable and could be modestly influenced by evidence-based risk estimates. The manuscript is well-written and relevant findings appropriately discussed. I have the following comments:

1. I would advise the authors to avoid beginning sentences in the results section of the abstract with a number.

Thank you – we changed this.

2. Although neurotrauma training is pretty standard across most residency programs, did the authors collect information about the type of practice for attending neurosurgeons? Were there differences based on type of fellowship training? Were spine surgeons more or less likely to recommend craniotomy? Did having additional neuro-critical care fellowship training influence treatment recommendation? If this information was not collected, the authors should mention the possibility of residual confounding due to such factors as one of the limitations of the results.

Because the study was randomized, there should be no confounding for the primary outcomes. However, the reviewer raises a very interesting question about the role of different practices/training types on prognostication. We added a comment about this in the limitation section and plan to address the issue in future work.

3. In the multivariable analysis presented in Table 4, I notice the authors did not adjust for other collected demographic characteristics like surgeon race/ethnicity, age and years of experience. Can the authors comment on that or provide a revised analysis adjusted for these covariates?

We added race, age and years of experience and updated the analyses.
